# Preoperative rectal cancer staging with phased-array MR

**DOI:** 10.1186/1748-717X-7-29

**Published:** 2012-03-05

**Authors:** Sabina Giusti, Piero Buccianti, Maura Castagna, Elena Fruzzetti, Silvia Fattori, Elisa Castelluccio, Davide Caramella, Carlo Bartolozzi

**Affiliations:** 1 Department of Diagnostic Radiology, University of Pisa, Via Roma 67, 56100-Pisa, Italy; 2 Department of Colorectal Surgery, University of Pisa, Via Paradisa 2, Cisanello, 56100-Pisa, Italy; 3 Department of Pathology, University of Pisa, Via Roma 67, 56100-Pisa, Italy

**Keywords:** Phased-array MRI, rectal cancer, mesorectal fascia, total mesorectal excision

## Abstract

**Background:**

We retrospectively reviewed magnetic resonance (MR) images of 96 patients with diagnosis of rectal cancer to evaluate tumour stage (T stage), involvement of mesorectal fascia (MRF), and nodal metastasis (N stage).

Our gold standard was histopathology.

**Methods:**

All studies were performed with 1.5-T MR system (Symphony; Siemens Medical System, Erlangen, Germany) by using a phased-array coil. Our population was subdivided into two groups: the first one, formed by patients at T1-T2-T3, N0, M0 stage, whose underwent MR before surgery; the second group included patients at Tx N1 M0 and T3-T4 Nx M0 stage, whose underwent preoperative MR before neoadjuvant chemoradiation therapy and again 4-6 wks after the end of the treatment for the re-staging of disease.

Our gold standard was histopathology.

**Results:**

MR showed 81% overall agreement with histological findings for T and N stage prediction; for T stage, this rate increased up to 95% for pts of group I (48/96), while for group II (48/96) it decreased to 75%.

Preoperative MR prediction of histologically involved MRF resulted very accurate (sensitivity 100%; specificity 100%) also after chemoradiation (sensitivity 100%; specificity 67%).

**Conclusions:**

Phased-array MRI was able to clearly estimate the entire mesorectal fat and surrounding pelvic structures resulting the ideal technique for local preoperative rectal cancer staging.

## Introduction

Rectal cancer is one of the most common tumour in Europe and in the United States (40 cases in every 100,000 individuals) [1] with a poor prognosis caused by high risk of local recurrence and metastasis. The local recurrence is related to the extramural tumour spread into the mesorectum and to the tumour distance from circumferential resection margin (CRM). Imaging plays a crucial role in the preoperative management of rectal carcinoma because traditional techniques usually performed to make diagnosis (colonoscopy and digital rectal examination), do not adequately show important prognostic features such as depth of tumour spread T stage) or the extent of lymph node involvement (N stage) [2,3].

At the present, the experts agreed that total mesorectal excision (TME) is the surgical approach of choice for rectal cancer, because it is able to reduce the local recurrence rate to less than 10% [4], improving the 5-year survival rate if compared with conventional surgery [5]. Moreover, after surgery local recurrence risk increases if the CRM is involved from the tumour. In selected patients with involvement of MRF at the time of diagnosis, the use of preoperative radiation therapy is advocated and it has been shown to further reduce the local recurrence rate from 8.2% to 2.4% at 2 years [5,6]. Using TME as surgical approach, MRF represents the CRM and consequently its involvement becomes the most important prognostic factor.

Due to this, preoperative staging techniques for rectal cancer should also distinguish patients with high risk of local recurrence and involvement of MRF, who might benefit from preoperative radiation therapy, from those patients at low risk, with the tumour located far from MRF, who might go directly to surgery. Then, it is always necessary to pay attention to the main risk factor for local recurrence that are: incomplete resection, nodal disease and distal tumour.

The aim of this report is to show that crucial role of phased-array MRI as technique of choice to preoperative locally stage rectal cancer, since it is the only technique able to accurately predict T and N stage and to depict the involvement of MRF.

## Materials and methods

### Study population

Between June 2005 - December 2008 we retrospectively reviewed 96 patients with histological diagnosis of rectal cancer, performed by endoscopical evaluation. This study population was subdivided into two groups: the first group was characterized by patients at T1-T2-T3 N0 M0 stage, whose underwent just a single MR examination before surgery. T3 stage patients, belonging to this group, despite the locally advanced disease, did not underwent neoadjuvant chemoradiation therapy, due to serious controindications to the treatment itself (such as chronic inflammatory disease, important diverticulitis, acute rheumatic disease, grave behaviour disease and the impossibility to maintain the treatment position). The second group included patients at Tx N1 M0 and T3-T4 Nx M0 stage of disease, whose underwent MR staging, then received neoadjuvant chemoradiation therapy and finally performed a second MR for the re-staging of disease.

**Figure 1:**
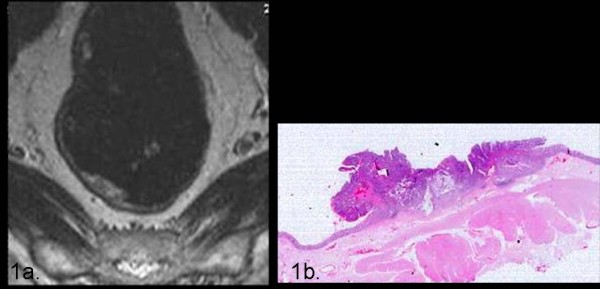
**The axial T2TSEwi sequence show a small T1 lesion infiltrating the right postero-lateral rectal wall with clear invasion of submucosa (1a) exactly correlating with the histology (1b)**.

**Figure 2:**
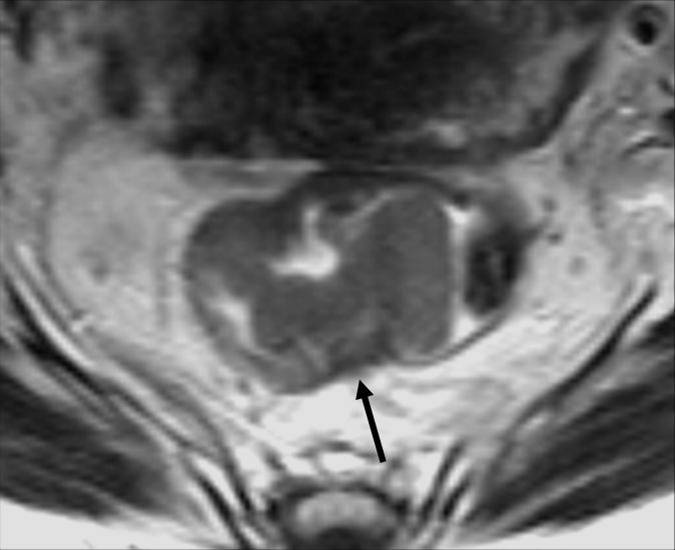
**The axial T2TSEwi sequence show a typical T2-stage lesion infiltrating muscolaris propria but limited to the bowel wall, which may be identified as the black line around the tumour**.

The lesions were considered as T1 stage tumours when clearly limited to submucosa (Figure 1a-b) and as T2 stage tumours when limited to the rectal wall (Figure 2). It is important to underline the not always it is possible to distinguish tumour growth limited to the submucosa or the invasion to the muscolaris externa: anyway, in these cases it is not necessary to make the always difference because both tumours are treated with TME-surgery (only in a minority of cases a T1 tumour will be treated with local excision and in these cases, when MR cannot distinguish between T1 versus T2 stage, endorectal US may help to identify superficial tumours).

**Figure 3:**
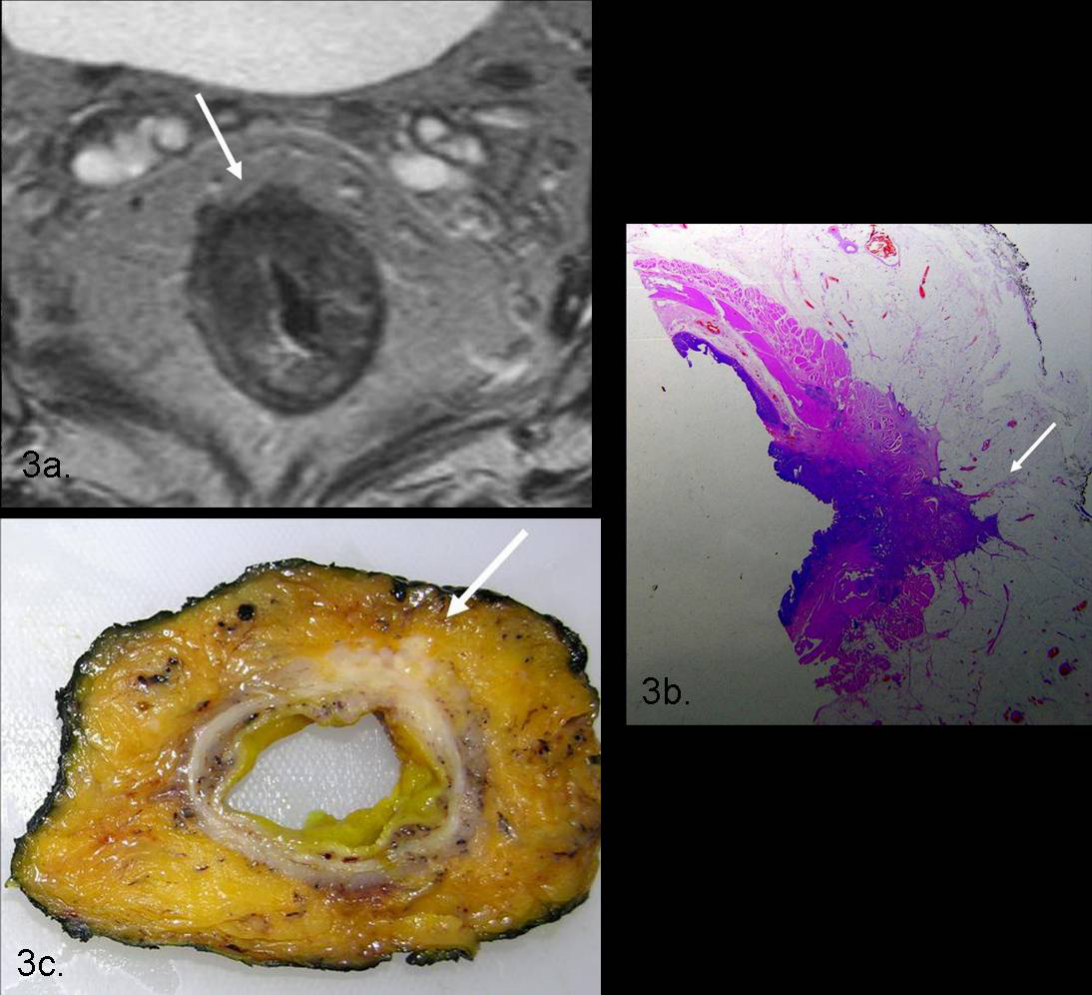
**The axial T2TSEwi sequence demonstrates a T3-stage tumour infiltrating perirectal fat**. There is a wide resection margin around the tumour and there are no lymphnodes adjacent to the MRF as demonstrated by the histology and by the corresponding gross specimen.

**Figure 4:**
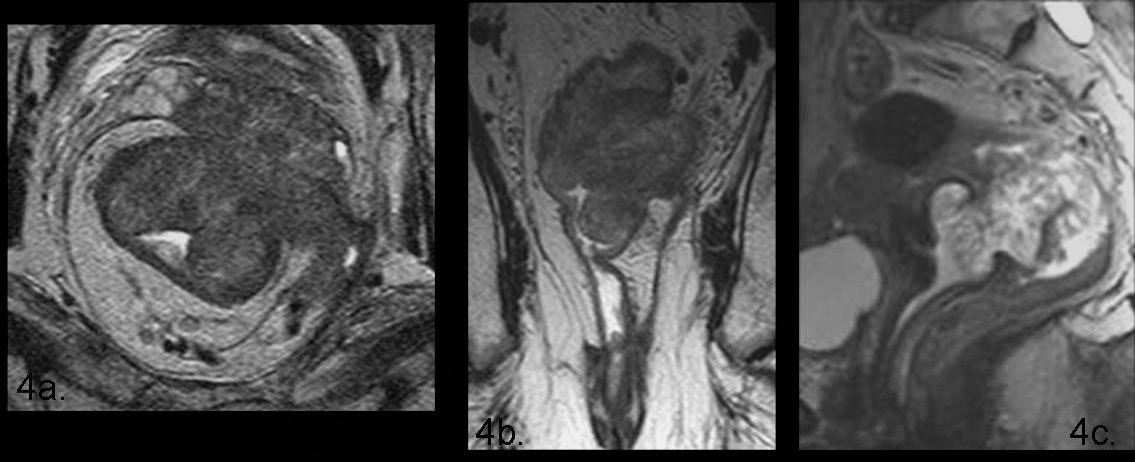
**In this picture there are some examples of T4-stage lesion invading surrounding structures such as seminal vescicle (4a: axial T2 TSE wi), left levator ani muscle (4b: coronal T2 TSE wi) and uterus (4c: sagittal T2 TSE wi)**.

T3 stage tumours were considered those lesions with a growth through all wall layers and extended into the perirectal fat tissue (Figure 3a-c) while T4 stage lesions showed an invasion of surrounding structures like pelvic wall, vagina, prostate, seminal vescicle or bladder (Figure 4a-b).

All MR findings were compared with histological results.

### MRI technique

MR imaging was performed with a 1.5- Siemens Medical System, Erlangen, Germany T MR imager (Symphony;) using a pelvic phased-array surface RF coil, necessary to reduce patient discomfort and to obtain higher spatial resolution images providing a full evaluation of rectal wall layers with the additional advantage of a large field of view.

All subjects were positioned in a supine, feet-first position with the lower edge of the coil placed at least 10 cm below the symphysis pubis in order to ensure adequate signal from the lower rectum and anorectal junction.

In our experience there was no need to use the endorectal coil because despite it may produce very high signal, its primary limitation is its small volume of sensitivity that is an insufficient anatomic detail from surrounding tissues to adequately stage rectal cancer. In fact, the area that can be imaged with endorectal coil amounts to a total distance of one coil diameter away from it that results in a very rapid drop in signal intensity beyond the immediate vicinity of the coil itself. Moreover, as with any other endoluminal technique, stenosis and stricturing, pain and discomfort, bowel wall motion, low lesions, lesions in the upper rectum and coil migration are all factors that hamper image acquisition.

No purgative bowel preparation or enemas, air-insufflation and contrast agents were administered. The use of intravenous antiperistaltic agents was not necessary to diminish movement in the small bowel since only the tumours of the lower two-thirds of the rectum were studied.

Multiplanar localizing images were obtained to select transverse, coronal and sagittal images with a T2- weighted fast spin-echo (FSE) sequence (repetition time msec/echo time msec, 4000-6000/100; 256 × 256 matrix, echo train length, 100-130; two signals acquired; sequence duration 3-5 minutes). These images were obtained with a 24-cm field of view and 4-mm-thick sections with no intersection gap. The sagittal images obtained were used to plan large field of view T2- weighted axial sections of the whole pelvis, from the iliac crest to the symphisis pubis (repetition time msec/echo time msec, 4000-6000/100; 256 × 256 matrix, echo train length, 100-130; two signals acquired; sequence duration 3-5 minutes). While the second series was being acquired, we again use the sagittal T2-weighted images obtained to plan to plan high-resolution T2-weighted thin-section axial images (repetition time msec/echo time msec, 4000-7000/100; 256 × 256 matrix, echo train length, 110-140; four signals acquired; sequence duration 5-7 minutes) through the rectal tumour and adjacent perirectal tissues. These images were performed perpendicularly to the long-axis of the tumour and rectal wall. The images were obtained by using a 16-cm field of view, a 3-mm section thickness and no intersection gap.

T1-weighted imaging (with/without contrast agent) was not performed, as it contributed any additional staging information to the analysis. No fat suppression was used to better delineate the tumour against the perirectal fat.

Some patients ( < 5%), either due to co-existing medical conditions or claustrophobia, found the scan impossible to tolerate. In these cases some of the sequences were omitted. Of all sequences the oblique high-resolution scans are the most important so the sagittal views is the only sequence which may be shortened by modifying the parameters and the large field of view. In these cases, axial T2-weighted images should be performed last and may be omitted if the patient is in considerable discomfort.

### Adjuvant therapy

Pre-operative neo-adjuvant therapy has been shown to improve overall survival in patient with rectal carcinoma [5] especially when it is added to TME surgery in terms of local control. There is a need to improve overall survival and this may be achieved by delivering systemically active chemotherapy synchronously with pre-operative radiation. Moreover, to protract the therapy help to downstage rectal tumours, reducing the rate of patients with positive CRM (CRM+). Sometimes (20% of cases) chemoradiation therapy lead to complete destruction of tumour cells [7].

The challenge is to identify pre-operatively patients with high risk of local recurrence performing TME surgery alone and hence identify who may benefit from preoperative chemo-radiation.

In our study we used high-resolution phased-array coil MRI to allow better selection of patients who could have required pre-operative neo-adjuvant therapy from those who could have be safely treated with surgery alone. 48/96 patients underwent 5 weeks of pre-operative chemo-radiation therapy using standard dose radiation (45 Gy in 25 daily fractions) concurrently with systemically effective chemotherapy.

Pre-operative MRI restaging was planned to take place 4-6 weeks after completion of neo-adjuvant therapy. Surgery was performed within 2 weeks following this pre-operative MRI restaging (6-8 weeks after completion of neo-adjuvant therapy).

### Specimen handlings and antomopathological technique

Good pathological reporting of rectal carcinoma is essential if we are to achieve the optimum possible results for patients with this tumour. There is nowadays good evidence that survival differs dramatically between surgeons and that the adoption of TME can improve local recurrence rates and survival [8-11].

After TME, each resected specimen was sent to the pathologist unopened and fresh (not in formalin). Not opening the specimen facilitates comparison with MRI which means an important quality control assessment for the radiologist. The specimen was photographed, prior and after inking, to allow audit of the quality of surgery. For a good resection the mesorectum should be smooth with no violation of the fat, good bulk to the mesorectum anteriorly and posteriorly and the distal margin should appear adequate with no coning near the tumour. No defect should be more than very superficial or 5 mm deep. Then the specimen was opened down to just above the tumour, but not through the tumour which was kept untouched to preserve assessment of the anterior aspect where CRM or peritoneal involvement may be seen. Anterior and posterior non-peritonealised surfaces were painted with ink.

After the resection surfaces were inked the specimen was fixed in formalin for a minimum of 3 days (72 hours). Good fixation allows thinner slices to be taken and thus a better assessment of tumour spread. The segment of the fixed specimen containing the tumour was then sectioned transversely at 5 mm intervals from 2 cm below to 2 cm above the tumour itself, producing slices that corresponded precisely to the MR sections.

Finally each slice was photographed and whole-mount histopathologic slices were cut and stained with hematoxylin-eosin. The extent of local tumor spread in each histopathologic slice was then assessed according to the TNM staging.

The local lymph nodes were identified and embedded, as should all the lymph nodes above and below the tumour.

The circumferential margin was considered involved if the tumour extended to within 1 mm of the circumferential excision margin.

## Results

The whole MR examination time was about 20 minutes, depending on the length of the tumour, and it was always well tolerated. Each MR image was interpreted by two experienced readers independently. The tumour, the layers of the rectal wall, the perirectal tissues and the mesorectal fascia were always well visualized in all patients (n = 96).

**Figure 5:**
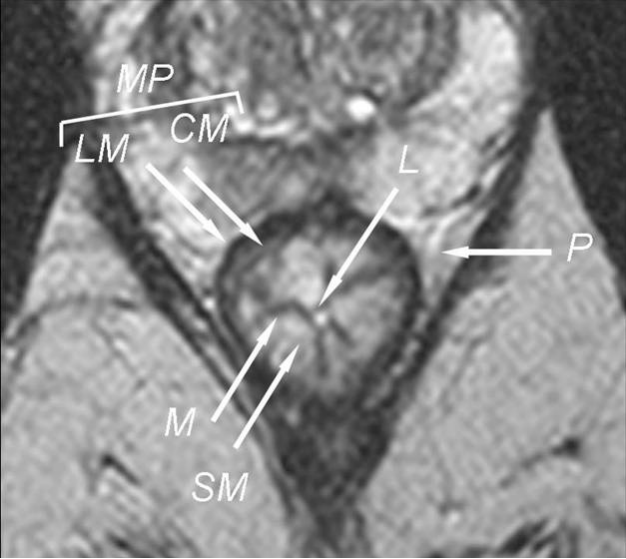
**Axial T2 TSEwi shows the three different layers of rectal wall: the inner hypointense and hyperintense layers respectively represent mucosa (M) and submucosa (SM), the hypointense inner and outer layers represent the muscolaris propria (MP) characterized by the inner circular muscle (CM) and the outer longitudinal muscle (LM); the external hyperintense layer represents perirectal fat tissue (P) while the internal the lumen (L)**.

The zonal anatomy of the rectal wall was best appreciated on thin-section axial images where the three different layers may be easily recognized: the inner hypointense and hyperintense layers respectively represent mucosa and submucosa, while the hypointense inner and outer layers represent the muscolaris propria characterized by the inner circular muscle and the outer longitudinal muscle; the external hyperintense layer represents perirectal fat tissue (Figure 5).

Even very thin hypointense structures such as the mesorectal fascia was always identified independent of the body habitus of the patient, owing to the high contrast between the hypointense fascia and the hyperintense fat tissue in and outside the mesorectum.

**Figure 6:**
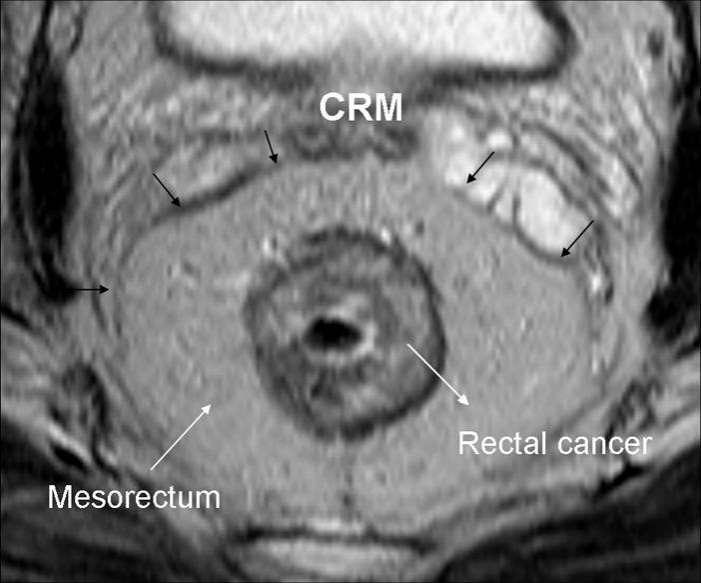
**Axial T2 TSEwi demonstrates a T2-stage rectal cancer bounded by the mesorectal fat and the mesorectal fascia; in particular the latter structure is appreciated on T2wi as a fine line of low signal intensity (black arrows)**. In a TME the mesorectal fascia correspond to the CRM.

Rectal carcinoma appeared as a higher signal intensity structure than rectal wall in all T2-weighted sequences so the infiltration was well appreciated thanks to the contrast between high-intermediate signal intensity of tumour and low signal intensity of surrounding muscle tissues. The fat tissue remained high in signal intensity and in this way, the tumour contrasted well also with the surrounding fat tissue (Figure 6).

**Figure 7:**
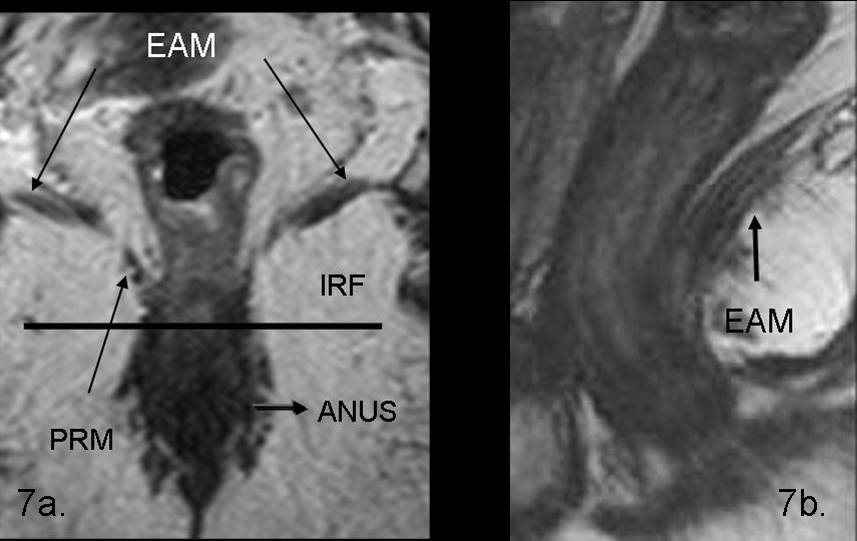
**These pictures show the normal rectal anatomy and the major anatomic structures such as the elevator ani muscle (EAM), the puborectal muscle (PRM), the internal and external anal sphincters, the ischio-rectal fossa (IRF) and the anal canal, easily evaluated on coronal T2 TSEwi (7a) and sagittal T2 TSEwi (7b)**.

Moreover, the major anatomic structures such as the levator ani muscle, the puborectal muscle, the internal and external anal sphincters, and the anal canal were easily evaluated (Figure 7a-b).

**Figure 8:**
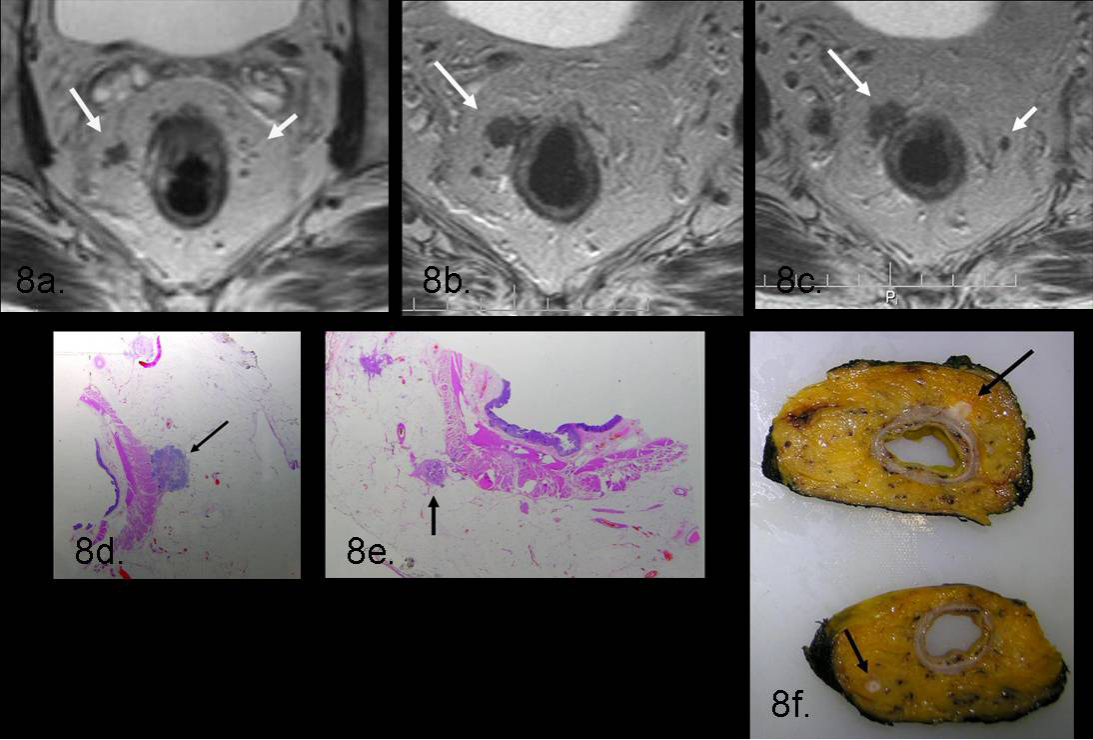
**In this picture it is represented on axial T2 TSE wi (8a) a T3-stage lesion infiltrating anterior rectal wall and perirectal fat; within mesorectum it is possible to appreciate multiple lymphnodes (white arrows in 8a-c)**. The hystolopathological slides (8d-e) and the corresponding slices of the gross specimen show the exact correlation with MR findings.

In the first group of pts MR demonstrated high accuracy in the prediction of correct T stage achieving an agreement rate with histology of 95%: in 2 cases, MRI overstaged attributing a T3 stage rather than a T2 (Table 1). Also for the identification of nodal disease (N stage) our diagnosis agreed accurately with histological data (94%) since MRI correctly assigned the N stage in 45/48 cases: in 2 patients MRI attributed a N1 stage while the histology did not confirm nodal disease; in 1 case MRI revealed no evidence of nodal involvement while histology reported N1 (Table 2) (Figure 8a-f).

**Table 1:** This table shows the high accuracy of MR examination to predict the correct T stage

AP	T1	T2	T3	T4	TOT
**MR**					
**T1**	**6**	0	0	0	6
**T2 **	0	**10 **	0	0	10
**T3**	0	2	**30 **	0	32
**T4**	0	0	0	**0**	0
**TOT**	6	12	30	0	**46**/48

In 2 cases, MR overstaged the lesions attributing a T3 stage rather than a T2. The agreement rate with histology was 95%.

**Table 2:** MRI correctly assigned the N stage in 45/48 cases: in 2 patients MRI attributed a N1 stage while the histology did not confirm nodal disease; in 1 case MRI revealed no evidence of nodal involvement while histology reported N1

AP	N0	N1	N2	N3	TOT
**MR**					
**N0**	**22**	1	0	0	23
**N1**	2	**13**	0	0	15
**N2**	0	0	**10**	0	10
**N3**	0	0	0	**0**	0
**TOT**	24	14	10	0	**45/**48

Agreement rate with histology resulted being 94%.

**Figure 9:**
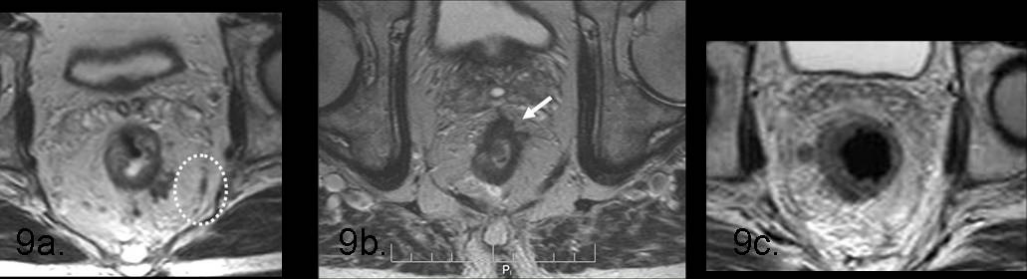
**In this picture there are represented different causes of mesorectal fascia infiltration: due to lymphangitic spread (9a); due to T3-tumour involving circumferential resection margin (9b); due to lymphnodes located within 1 mm of the mesorectal fascia (9c)**. In particular, this last case suggest that whenever there are lymphnodes located strictly near the mesorectal fascia, especially when they are large, we need to report this because the circumferential resection margin may be involved.

MRI with phased-array coil showed high accuracy in prediction of a tumour-involved MRF (MRF+) with a sensibility and a specificity of 100% (Table 3) (Figure 9a-c).

**Table 3:** The table shows the high accuracy of MR in prediction the involvement of mesorectal fascia (MRF+): sensitivity and specificity resulted both 100%

AP	MRF+	MRF-	TOT
**MR**			
**MRF+**	**4**	0	4
**MRF-**	0	**44**	44
**TOT**	4	44	48

Preoperative chemo-radiotherapy was administered to patients belonging to group II with locally advanced rectal cancer and then treated with TME. Locally advanced rectal cancer was defined as tumour extension through the bowel wall, based on clinical and MR evaluation, without associated distant metastases.

In this second group population of patients submitted to neoadjuvant therapy MRI correctly assigned tumour stage (T) in 36/48 cases with 75% of agreement with the histological results. In 12 cases MRI overstaged: 2 patients were staged as T2 rather than T1; 8 cases were staged as T3 rather than T2; 2 lesions were classified as T4 but resulted T3 at histology (Table 4).

**Table 4:** The table shows that MR correctly assigned tumour stage in 36/48 cases with 75% of agreement with the histological results

AP	T1	T2	T3	T4	TOT
**MR**					
**T1**	10	0	0	0	10
**T2**	2	**8**	0	0	10
**T3**	0	8	**14**	0	22
**T4**	0	0	2	**4**	6
**TOT**	12	16	16	4	3**6***/*48

12 cases were overstaged: 2 patients were staged as T2 rather than T1; 8 cases were staged as T3 rather than T2; 2 lesions were classified as T4 but resulted T3 at histology.

MRI showed a sensibility of 100% and a specificity of 67% in prediction of a tumour-involved MRF (MRF+) as shown in table 5.

**Table 5:** The table shows the high accuracy (92%) of MR for nodal metastasis: 2 cases were overstaged ( N1 stage rather than N0) and 1 in case MR did not reveal any nodal involvement whereas histology reported N1

AP	N0	N1	N2	N3	TOT
**MR**					
**N0**	**22**	1	0	0	23
**N1**	2	**13**	0	0	15
**N2**	0	0	**10**	0	10
**N3**	0	0	0	**0**	0
**TOT**	24	14	10	0	**45*/***48

For nodal metastasis (N stage) MRI showed 92% accuracy with 2 cases of overstaging ( N1 stage rather than N0) and in 1 case MRI did not reveal any nodal involvement whereas histology reported N1 (Table 6).

**Table 6:** The table show the high sensitivity (100%) and specificity (67%) of MR in predicting MRF+

AP	MRF+	MRF-	TOT
**MR**			
**MRF+**	**12**	12	24
**MRF-**	0	**24**	**2**4
**TOT**	12	36	48

**Figure 10:**
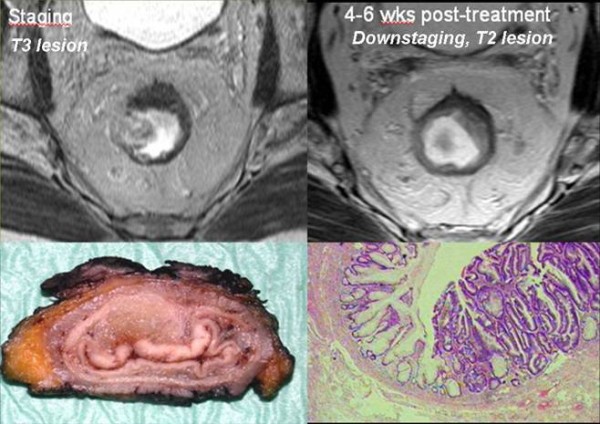
**This picture shows a T3 tumour infiltrating the rectal wall and perirectal fat, with some irregular and heterogeneous lymph nodes within the mesorectum**. After neo-adjuvant therapy there is an evident tumour regression without any invasion into the surrounding tissues as demonstrating by the gross specimen, where only a gelatinous mash has remained inside the rectal wall, and by the corresponding histological slice where any evidence of extraluminal infiltration is present anymore.

Moreover, in this second group of patients phased-array MRI assessed also the treatment outcome. In fact, 22/48 cases resulted downstaged: 2 rectal cancer staged as T4 at preoperative MR became T3 lesions after chemoradiation; 14 cases staged as T3 have became T2 (Figure 10); 4 case staged as T3 downstaged to T1 and 2 cases staged as T2 resulted T1 at the end of the treatment.

## Discussions

Our study demonstrated the high accuracy of preoperative MRI in the prediction of correct T stage; the agreement with histopatholgy was about 95% and it correlated with the data reported in the most of the studies published in literature (65%-100%) [12-16].

For T staging, results of accuracy of phased-array coil have been demonstrated to be similar to those obtained with an endorectal coil, but with the advantage that also nodal staging and a detailed imaging of all anatomical structures above the pelvic floor is possible.

Moreover, there is no patient discomfort because no insertion of any rectal coil is needed: the patient have merely just to lie on the MR table and his/her comfort is further enhanced in our technique by using the feet-first supine position, resulting in fewer problems with claustrophobia.

MRI demonstrated high performance also to assign the correct tumour stage of small lesions confined to rectal wall: T1 (16/20) and T2 (18/28).

The most frequent diagnostic error caused by MRI consisted in correctly differentiated T2 from early T3 lesions: this overstaging was often caused by the presence of desmoplastic reaction within the peritumoral tissues that made difficult the MR differentiation between perirectal fat spiculation, caused by fibrosis alone from those containing viable tumour cells.

The major problems occurred when MRI was performed after neoadjuvant therapy because the agreement rate with histhological results comes down from 95 to 75% due to post-radiation oedema, inflammation, fibrosis and necrosis that made difficult a correct diagnosis.

As far as concern the prediction of MRF tumour invasion, MRI was extremely accurate achieving a sensibility and a specificity of 100% in the first group. Also for this aspect our results were in accordance with those published in literature, but decreased after neoadjuvant therapy (II group) (specificity, 67%).

A recent Dutch study introduced a profile very similar to our study as far as concerns the population of patients that performed neoadjuvant therapy (48/96); our data were indeed comparable with those (sensibility of 100%, specificity of 32-59%): in both cases the lowest values of specificity came from the overstaging. In the most of patients when disease was overstaged at MRI, a diffuse hypointense tissue infiltrating MRF, corresponded to sterilized areas of fibrosis at histological analysis. Indeed postchemoradiation MRI was not able to discriminate between the fibrotic spiculations in the perirectal fat and spiculations with contextually malignant cells at histological analysis [17].

The performances of MRI in the local staging of rectal cancer disease confirm the possibility of this technique to discriminate patients candidated to preoperative neoadjuvant therapy especially considering the high performances of MRI in defining MRF involvement. A recent trial reported that, in selected patients, with FMR+ at the moment of the diagnosis, the use of the preoperative radiation therapy is mandatory because reduces the recurrence rate from 8.2% to 2.4% to 2 years [18].

In our experience, radiologist can be sure that the MRF will be free, only when an intact rectal wall is detected.

In this respect, the present T-staging system have its shortcomings because it does not discriminate between cancers with a wide FMR and cancers with a close or involved FMR. Although most of those tumours are classified as stage T3, they have a different risk of local recurrence. In fact today it's unanimously agreed that the distance of the cancer from the CRM, represented by the MRF, is a more powerful predictor for the local recurrence rate, much more significant than the T stage. In particular, if the distance of the tumour from MRF is ≥ 5 mm, radiologist may consider the fascia uninvolved; if the distance is 2-5 mm the involvement is considered border-line; if it is ≤ 1 mm, MRF is involved.

As regard N stage, identification of nodal disease is still a diagnostic problem for the radiologist. Despite the identification of lymph nodes as small as 2-3 mm on high-spatial-resolution images, reliable detection of nodal metastasis presently is not possible. The radiologist assessment of nodal involvement generally relies on morphologic criteria such as the size and shape of the node. The problem with morphologic imaging, however, is that with enlarged nodes is difficult to distinguish between reactive and metastatic nodes, and with small nodes micrometastases are easily missed. An additional problem in rectal cancer is the high frequency of micrometastases in normal-sized nodes. In fact, there is currently no consensus about lymph node size vis-à-vis nodal involvement: some authors report any detectable lymph node, whereas others report only those lymph nodes that are larger than a given size (3 mm, 5 mm, or 10 mm) [19].

In our study nodal evaluation had been estimated with dimensional cut-off of 10 mm achieving an agreement rate with histology of 96% that was comparable with the values of literature but it is obvious that also our approach has been arbitrary.

However, results of early anatomic studies showed that over half of the metastatic nodes were within 3 cm of the primary tumour and were smaller than 5 mm in size [20-25]; these data evidently call in question the real validity of the dimensional criteria.

Our experience has confirmed that MR with phased-array coil is the technique of choice in the preoperative local staging of rectal cancer since it helps to estimate in a detailed and non-invasive way the entire mesorectal fat and all the surrounding pelvic structures, MRF included; moreover, it allows to identify the different risk group of patients helping to decide about the treatment and its outcome (downstaging with complete or incomplete response), and finally to optimize a complete excision allowing to those patients with an advantaced rectal cancer to benefit from a sphincter-preserving surgery.

## Competing interests

The authors declare that they have no competing interests.

## Authors' contributions

SG: carried out the study and drafted the manuscript. PB: performed surgery. MC: performed anatomopathological evaluation. EF: participated in the design of the study. SF: participated in the anatomopathological evaluation. EC: participated in the anatomopathological evaluation. DC: coordinated the study. CB: coordinated the study and participate in its design. All authors read and approved the final manuscript
